# Role of Vitamin D Beyond the Skeletal Function: A Review of the Molecular and Clinical Studies

**DOI:** 10.3390/ijms19061618

**Published:** 2018-05-30

**Authors:** Meenakshi Umar, Konduru S. Sastry, Aouatef I. Chouchane

**Affiliations:** Division of Translational Medicine, Research Department, Sidra Medicine, P.O. Box 26999 Doha, Qatar; meenakshiumar@gmail.com (M.U.); skonduru@sidra.org (K.S.S.)

**Keywords:** vitamin D, non-phosphocalcic action, cellular functions, immune regulation, clinical effect

## Abstract

The classical function of Vitamin D, which involves mineral balance and skeletal maintenance, has been known for many years. With the discovery of vitamin D receptors in various tissues, several other biological functions of vitamin D are increasingly recognized and its role in many human diseases like cancer, diabetes, hypertension, cardiovascular, and autoimmune and dermatological diseases is being extensively explored. The non-classical function of vitamin D involves regulation of cellular proliferation, differentiation, apoptosis, and innate and adaptive immunity. In this review, we discuss and summarize the latest findings on the non-classical functions of vitamin D at the cellular/molecular level and its role in complex human diseases.

## 1. Introduction

Vitamin D is a secosteroid whose function was long considered to be the maintenance of bones and calcium/phosphorous homeostasis. In the last few decades, the extra-skeletal effects of vitamin D became apparent and its effect on the cellular proliferation, differentiation, and immune modulation has been profoundly investigated. Furthermore, reports of protective effects from vitamin D in several diseases like hypertension, diabetes, cardiovascular diseases, autoimmune diseases, and cancers indicate a significant upturn in its role beyond the well-known anti-rachitic factor [1]. In this review, we will comprehensively review the non-classical function of vitamin D with emphasis on its molecular and clinical aspects.

## 2. Vitamin D: Sources and Metabolism

Vitamin D occurs in two major forms, which includes vitamin D2 (ergocalciferol) and vitamin D3 (cholecalciferol). Vitamin D2 is synthesized by ultraviolet B (UVB) irradiation of the ergosterol found in yeast and fungi and it is present in a small number of natural foods (such as UVB-radiated mushrooms), in fortified food, and supplements [2]. On the other hand, vitamin D3 is obtained by photochemical reaction in the skin and through diet via intake of animal-based foods (like cod liver oil and oily fish).

The major source of vitamin D is UVB radiation-induced photochemical and thermal conversion of 7-dehydrocholestrol in the skin. Absorption of UVB radiation (290–315 nm) in the skin results in the opening of ring B of 7-dehydrocholestrol, which forms a thermodynamically unstable pre-vitamin D3 (9,10-secosterol). This thermally isomerizes into more stable vitamin D3 (cholecalciferol). Vitamin D either formed in the skin or was absorbed from the diet in the small intestine and was transported to the liver. In the liver, vitamin D is hydroxylated by vitamin D 25-hydroxylase (CYP2R1) to produce 25-hydroxy vitamin D (25(OH)D) or calcidiol, which is the accepted biomarker for vitamin D status [3,4]. 25(OH)D is then transported to the kidneys where it is further hydroxylated by 1-α-hydroxylase (CYP27B1) to produce its biologically active form 1-α-25-dihydroxy vitamin D (1,25(OH)2D, also known as calcitriol). Vitamin D and its metabolites are transported to the target cells primarily by binding to the vitamin D binding protein (DBP). Furthermore, 25(OH)D and 1,25(OH)_2_D are metabolically inactivated through hydroxylation by 24-hydroxylase (CYP24A1). The vitamin D level in serum is closely regulated through feedback loops involving the actions of calcium, phosphorous, 1,25(OH)_2_D, parathyroid hormone (PTH), and fibroblast growth factor 3 [5,6]. According to the guidelines of US Endocrine Society, the serum levels of 25(OH)D below 20 ng/mL (50 nmol/L) are stated as vitamin D deficiency while 25(OH)D serum levels between 21–29 ng/mL (52.5–72.5 nmol/L) are defined as vitamin D insufficiency [7]. For subjects with serum 25(OH)D levels between 20 and 21 ng/mL, it is generally accepted that values of serum 25(OH)D levels below 20.5 ng/mL are taken as 20 ng/mL and, therefore, considered vitamin D deficiency while serum 25(OH)D levels ≥ 20.5 ng/mL are taken as 21 ng/mL and considered to be vitamin D insufficient.

## 3. Non-Phosphocalcic Action of Vitamin D

For many years, the function of vitamin D pro-hormone was considered to be limited to calcium and phosphorus homeostasis. However, after the discovery of vitamin D receptors (VDR) in various cell types (like keratinocytes, lymphocytes, parathyroid and pituitary gland cells, pancreatic cells, etc.), many biological roles of vitamin D have been revealed in addition to its known actions in classic target tissues [8,9]. The non-classical function of vitamin D includes the regulation of several physiological processes like cell proliferation, differentiation, and immune modulation. Vitamin D mediates its function by binding to the VDR, which is a member of nuclear hormone receptors superfamily. VDR activated by vitamin D interacts with retinoid X receptor to form a heterodimeric complex, which is recruited to the vitamin D response elements (VDRE) in the target genes to activate or to repress their expression through interaction with additional co-regulators.

### 3.1. Cellular Proliferation and Differentiation

Vitamin D affects the cellular proliferation by modulating different processes including apoptosis, cell cycle progression, and differentiation in a cell specific manner. It can affect the cell proliferation either directly through binding of VDR (activated by ligand binding) to the response element in the genes regulating cell growth or indirectly by influencing key transcriptional regulators or cell signaling molecules involved in apoptosis, the cell cycle, and differentiation.

#### 3.1.1. Cell Cycle

Vitamin D is most commonly reported to repress cell cycle progression by causing cell cycle arrest at G0–G1 transition in a cell specific manner. In many cell lines, 1,25(OH)_2_D3 induced cyclin dependent kinases inhibitors (CDKIs) expression like p27kip1 and p21WAF1/CIP1 to mediate cell cycle arrest [10,11,12,13,14]. Vitamin D compounds are also reported to alter other regulatory proteins of the cell cycle like INK4, p53, and p21-activated kinase. In addition, calcitriol was found to decrease cyclin D1 and cyclin D3 levels in the MCF-7 breast cancer cell line [15,16] and cyclin E expression in MG-63 osteosarcoma cell line [17], which, in turn, caused inhibition of CDK activity and retinoblastoma protein hypo-phosphorylation. Contrary to the widely reported cell cycle arrest at G0–G1 stage, two reports suggested that vitamin D can mediate cell cycle arrest at later stages: at the G2/M stage by induction of GADD45a in ovarian cancer cell lines and at the G1 stage in the human adrenocortical carcinoma cell line H295R [18,19].

#### 3.1.2. Differentiation

Vitamin D and its analogs regulate the differentiation of many malignant/benign tumor cells and also normal cells. Abe et al. reported that 1,25(OH)_2_D3 stimulates the differentiation of cultured mouse myeloid leukemia cells to macrophages [20]. Similarly, Halline et al., showed that 1,25(OH)_2_D3 promotes the differentiation and stimulates the formation and maturation of an apical microvillus membrane in human intestinal Caco-2 cells [21]. EB1089, which is a vitamin D analog, promoted the differentiation and reversed the malignant phenotype of squamous carcinoma cells, SCC25, by inducing several differentiation-promoting epithelial genes (like *Cystatin M*, *Protease M*, *Type XIII collagen*, and *Desmoglein 3*) [22]. Similarly, vitamin D compounds showed an inhibitory effect ontriple negative breast cancer by downregulating key markers of breast cancer stem cells and by upregulating myoepithelial differentiating markers (cytokeratin 14 and smooth muscle actin) [23]. In cardiac cells and colorectal micro-adenoma, vitamin D promoted differentiation through inhibition of Wnt signaling [24,25].

#### 3.1.3. Apoptosis

Vitamin D can modulate key mediators of apoptosis in many cancer cell lines and normal cells. Vitamin D triggers apoptosis mainly by inhibiting the anti-apoptotic proteins and/or by stimulating the pro-apoptotic proteins. In human squamous cell carcinoma, inecalcitol (a vitamin D analog) induced apoptosis by activating the caspase 8/10-caspase 3 pathway and by inhibiting anti-apoptotic proteins like cellular inhibitor of apoptosis protein-1 (c-IAP1) and X-linked inhibitor of apoptosis protein (XIAP) [26]. However, 1,25(OH)_2_D3 lowered the expression of B-cell lymphoma (BCL) protein 2 (BCL2) and B-cell lymphoma-extra large (BCL-X_L_) mRNA and increased the expression of Bcl-2-associated X (BAX) and p21 mRNA to promote apoptosis of the chronic myeloid leukemia cell line K562 [27]. Similarly, Diaz et al., showed that the vitamin D analog EB1089, through upregulation of BCL2 antagonist killer (BAK), stimulates apoptosis in many colon cancer cell lines [28]. In human retinoblastoma-derived Y79 cells, 1,25(OH)_2_D3 and its synthetic analog KH1060 promoted apoptosis by increasing the BAX protein and reducing BCL2 protein levels [29]. Vitamin D can also trigger apoptosis by mechanisms other than the action on the BCL2 protein family. Sergeev et al., showed that 1,25(OH)_2_D3 induces Ca^2+^ signal in breast cancer cells and adipocytes, which directly recruits Ca^2+^-dependent apoptotic effectors, calpain, and caspase 12 to initiate apoptosis [30]. In another study, vitamin D analogs EB1089 and CB1093 promoted apoptosis in breast cancer cells by suppressing insulin-like growth factor 1 (*IGF1)* signaling [31]. In kidney cancer cells, it has been shown that vitamin D3 promotes apoptosis by activating forkhead box O3 (FOXO3) through downregulation of phosphorylated serine-threonine protein kinase Akt and extracellular signal-regulated kinase (Erk) [32]. In contrast to the above literature, few reports suggested an inhibitory effect of vitamin D on apoptosis [33,34].

### 3.2. Vitamin D and Immune Regulation

The immune-modulatory action of vitamin D originated from two main observations: (i) the presence of VDR in proliferating immune cells and (ii) the ability of immune cells to metabolize vitamin D [35]. The latter function ensures a physiological high concentration of active 1,25(OH)_2_D3 in a local lymphoid environment, which promotes its specific action and limits any undesirable high concentration-related systemic effects like hypercalcemia and bone resorption [36]. Locally produced vitamin D acts on immune cells either in intracrine, autocrine, and/or paracrine fashion and affects multiple components of innate and adaptive immunity pathways.

#### 3.2.1. Vitamin D and Innate Immunity

Vitamin D affects this component of the immune system through its action on anti-microbial peptides synthesis and antigen presentation.

##### Synthesis of Anti-Microbial Peptides (AMP)

Antimicrobial peptides are low molecular weight host defense peptides with a broad spectrum antimicrobial activity against bacteria, viruses, and fungi. Cathelicidin and defensin are two major groups of epidermal anti-microbial peptides (AMPs), which are reported to be induced by vitamin D in the immune cells and in a variety of other cells outside the classical immune system. Wang et al., showed that treatment of 1,25(OH)_2_D3 leads to robust induction of cathelicidin in neutrophils, monocytes, human keratinocytes, SCC25 (head and neck squamous carcinoma cells), Calu-3 (lung adenocarcinoma cells), and U937 (myelomonocytic cells) [37]. Similarly, in another study, 1,25(OH)_2_D3 and its analogs were reported to induce cathelicidin expression in acute myeloid leukemia (AML) cell line, keratinocyte, colon cancer cell lines, and in macrophages derived from bone marrow of AML patients/controls [38]. It was also shown that vitamin D moderately increases the expression of another AMP β-defensin 2 in some human cell lines (like SCC25, Calu-3 cells, and primary cultures of adult keratinocyte) and this effect is enhanced in the presence of interleukin 1 (IL-1) [37]. Cathelicidin is a direct transcriptional target of vitamin D, which is induced by binding of 1,25(OH)_2_D3-VDR complex to the VDRE in the promoter of the gene. However, β-defensin 2 requires nuclear kappa B (NF-κB) along with 1,25(OH)_2_D3-VDR complex for its transcription [39].

##### Antigen Presentation

Antigen presenting cells (APC) of the innate immune system stimulate the lymphocytes of adaptive immunity through antigen presentation to remove the infectious agents. Dendritic cells (DCs) are the most potent APC and are broadly classified into two subtypes based on their origin including myeloid DCs (mDCs) and plasmacytoid DCs (pDCs). 1,25(OH)_2_D3 and its analogs are reported to inhibit the maturation, differentiation, and survival of DCs [40,41]. In addition, studies showed that treatment of 1,25(OH)_2_D3 inhibits the antigen presentation by DCs and primes cells towards tolerogenic state [42,43]. In accordance with this, Penna et al., showed that treatment of 1,25(OH)_2_D3 maintains the immature phenotype of DCs (marked by low mannose receptors and low CD38 expression) and prevents stimulation of co-stimulatory molecules (CD40, CD80, and CD86) and major histocompatibility complex (MHC) class II protein expression in DCs by reducing its capacity to activate alloreactive T cells [44]. Vitamin D3 also inhibits immune-stimulatory cytokine IL-12 secretion [45] and increases the production of immune-suppressive cytokine IL-10 by DCs [44]. The overall effect of vitamin D treatment on DC is the decrease in T helper 1 (Th1) cell response. The induction of IL-10 produces regulatory T (Treg) cells and promotes immune tolerance. Studies investigating the effect of vitamin D on DCs subtypes showed that it selectively induces tolerogenic properties in mDCs despite comparable VDR signal transduction in both subtypes [46,47].

#### 3.2.2. Vitamin D and Adaptive Immunity

The adaptive immune system shows an antigen-specific immune response and mediates its effect via T and B cells. Early studies have demonstrated the expression of VDR in B and T lymphocytes particularly in an immunologically active state [48,49,50]. Vitamin D can have either an indirect effect on lymphocytes through paracrine signaling by APC (as discussed earlier) or a direct effect by VDR signaling. Several studies indicated that 1,25(OH)_2_D3 suppresses T lymphocytes proliferation most likely by reducing IL-2 transcription [51,52,53,54]. The effect of vitamin D on different components of adaptive immunity is described in the following sections.

##### CD4^+^ T Cells

CD4^+^ T cells, also known as T helper (Th) cells, recognize peptides presented by MHC Class II molecules of APC. Based on the pattern of cytokines secreted, they are classified into Th1, Th2, recently identified Th17 cells, Th22, and Treg cells. In many in-vitro studies, 1,25(OH)_2_D3 inhibited the secretion of interferon gamma (IFN-γ) by CD4^+^ T cells including Th1 cells [55,56,57,58]. Vitamin D3 was also reported to stimulate the development of Th2 cells mainly by increasing the synthesis of IL-4 [57,58,59]. However, few studies have observed either no effect or repressive effect on IL-4 secretion by vitamin D3 [60,61,62]. It has also been shown that 1,25(OH)_2_D3 reduces the expression of IL-17, which is the prototype cytokine of Th17 cells [56,60,63,64]. The production of IL-22 (which may be a product of Th17 cells or Th22 cells) is also decreased by 1,25(OH)_2_D3 [63,65]. Similarly, a study showed that oral intake of cholecalciferol (daily escalation starting from 2000 up to 8000 IU daily for 12 weeks) resulted in significantly decreased frequencies of IFN-γ^+^ and/or IL-17^+^ CD4^+^ Th cells [66].

##### CD8^+^ T Cells

CD8^+^ T cells, also known as cytotoxic T cells, recognize the peptides presented by MHC class I molecules, which are present on all nucleated cells. CD8^+^ T cells have the highest level of VDR expression compared to other immune cells [49]. Vitamin D3 reduces the proliferation of CD8^+^ T cells. VDR knockout (KO) CD8^+^ T cells exhibit an increased proliferation without antigen stimulation because of an increased production of IL-2 [61,67,68]. Vitamin D3 deficiency is supposed to augment the gastrointestinal inflammation through its effect on CD8αα cells. Yu et al., showed that VDR KO mice present with a decreased number of CD8αα cells (with low level of IL-10) and with an increased inflammatory response [69]. Some studies have investigated the influence of vitamin D3 on the expression of cytokines secreted by CD8^+^ T cells like IL-6, IL-12, tumor necrosis factor alpha (TNF-α), IL-5, and transforming growth factor beta (TGF-β). Willheim et al., suggested an increased frequency of IL-6 positive and a reduced number of IL-12 positive CD8^+^ T cells in cultures with 1,25(OH)_2_D3 [61]. In addition, Lysandropoulos et al., showed that 1,25(OH)_2_D3- treated CD8^+^ T cells secret less IFN-γ and TNF-α and more IL-5 and TGF-β [70].

##### Regulatory T (Treg) cells

Treg cells are a subset of T cells that suppress the immune response and mediate immune tolerance. These cells are most widely characterized either as naturally occurring CD4^+^CD25^+^ T cells (associated with Forkhead box P3 (Foxp3) transcription factor) or as antigen-driven inducible Treg cells (iTreg). iTreg cells are further categorized into IL-10-producing Treg (Tr1) and TGF-β secreting Treg (Th3) cells [71]. 1,25(OH)_2_D3 not only enhances IL-10 secretion by CD4^+^ T cells but also increases the frequency of IL-10 producing Treg cells [57,60,62,72]. Similarly, 1,25(OH)_2_D3 treatment was shown to promote the development of Foxp3^+^CD4^+^ T cells [56,73,74]. In addition, a strong positive correlation was found between Foxp3^+^ T cells and the serum 25(OH)D level in patients with asthma, multiple sclerosis, and Behcet disease, which points to the role of vitamin D in the induction or activation of Treg cells [75,76,77].

##### Natural Killer T (NKT) Cells

NKT cells are specialized T lymphocytes that play an important role in autoimmunity, cancer, and infections. They are initial up-regulators of IL-4, IFN-γ, and other cytokines during infection [78]. Studies showed that VDR and its ligand vitamin D regulate the normal development and function of invariant NKT cells (iNKT, a variant of NKT cells) [79,80]. It was also observed that the iNKT cells isolated from VDR knock out mice do not function well and secret less IL-4 and IFN-γ [79]. Waddell et al., suggested that 1,25(OH)_2_D3 mediates a protective effect in autoimmune encephalomyelitis (EAE) through NKT [81]. He demonstrated that 1,25(OH)2D3 treatment failed to prevent the development of EAE in the NKT knock out mice model while it protected wild type mice (which develops EAE on control diet) from developing EAE by affecting NKT specific IL-4 secretion [81].

##### B Cells

B cells are antibody-secreting cells of the immune system. Resting B cells express very low levels of VDR, which is upregulated upon activation with various stimuli [82,83,84]. 1,25(OH)_2_D3 inhibits the proliferation and promotes the apoptosis of activated B cells [82,85]. Additionally, 1,25(OH)_2_D3 was observed to inhibit plasma cell generation and memory B cell formation [82]. Subsequently, the secretion of immunoglobulins IgG and IgM by activated B cells is also shown to be inhibited by vitamin D3 treatment in many in-vitro studies [82,83,84,85]. Experimental studies suggest that the calcitriol treatment can reduce IgE synthesis by activated B cells and can lower serum IgE level in the type 1 allergy mouse model [86,87]. However, observational studies on the relationship between vitamin D3 and IgE conflict. Certain studies showed an inverse relationship between serum IgE and 25(OH)_2_D3 levels while others showed a positive correlation. In addition, a nonlinear U-shaped association between vitamin D and serum IgE levels was suggested by other experts [88,89,90,91]. Vitamin D also regulates another relatively small subset of B cells known as regulatory B (Breg) cells. Breg cells are involved in immunological tolerance by producing IL-10, IL-35, and TGF-β cytokines. In an in-vitro study, 1,25(OH)_2_D3 treatment significantly augmented the production of IL-10 by activating B cells [92]. However, in a cross-sectional study, no correlation was observed between vitamin D status and the frequency of Breg cells in multiple sclerosis patients [93].

## 4. Molecular and Clinical Studies of Vitamin D in Various Diseases

The widespread effect of vitamin D on different physiological processes and the association of vitamin D deficiency to various disorders point towards the potential therapeutic role of this molecule. A randomized double blind clinical trial showed that vitamin D supplementation in vitamin D-deficient subjects significantly affects the expression of genes involved in pathways linked to cancer, auto-immune disorders, and cardiovascular diseases [94]. In this section, we will summarize the findings of several molecular and clinical studies by investigating the role of vitamin D in diverse health disorders.

### 4.1. Cancer

Vitamin D exerts a beneficial effect on cancer by inhibiting the proliferation, angiogenesis, and metastasis of cancer cells and by promoting the differentiation and apoptosis of these cells. Li et al., showed that vitamin D3 inhibits the proliferation of gastric cancer cells by stimulating p21 and suppressing CDK2 [95]. Similarly, in melanoma cell lines, vitamin D3 treatment mediated an anti-proliferative effect and modulated the expression of key cell cycle regulatory molecules like p21, p27, cyclin D1, and cyclin A1 [96]. The anti-metastatic activities of vitamin D is thought to be mediated by its ability to downregulate the proteases that promote the degradation of the extracellular matrix (like matrix metalloproteinases (MMP9 and MMP13), and cathepsin) and by its capacity to upregulate the protease inhibitors (like tissue inhibitor of metalloproteinase 1 (TIMP-1) and cathepsin inhibitor) that inhibit the degradation of the extracellular matrix [97,98]. Vitamin D is reported to regulate angiogenesis in several ways [99] including several in vitro and in vivo studies that have shown calcitriol downregulates proangiogenic factors like Hypoxia inducible factor 1 and its response proteins including vascular endothelial growth factor (VEGF) in many cancers [100,101]. Another way vitamin D regulates angiogenesis is through NF-κB signaling, which induces angiogenic factors (like IL-8 and VEGF) and was suppressed by vitamin D in prostate cancer cells [102]. Additionally, it was demonstrated that calcitriol may inhibit angiogenesis in some cancers through suppression of prostaglandin pathways [103]. Recently, vitamin D compounds have also been shown to inhibit cancer stem cells by down regulating cancer stem cell markers (like *OCT4*, *CD44*, and *LAMA5*) and by inhibiting Notch signaling molecules (involved in cancer stem cell maintenance). This suggests an additional mechanism through which vitamin D mediates its beneficial role in cancer metastasis and treatment resistance [23,104]. The pro-apoptotic and pro-differentiating actions of vitamin D in cancer cells are discussed in detail in Section 3.1.2 and Section 3.1.3.

Similar to experimental studies, most of the epidemiological studies have suggested a beneficial effect of vitamin D in cancer risk, incidence, and mortality. A data from pooled analysis of randomized clinical trials (RCT) and prospective cohort studies suggested that 25(OH)D serum concentrations ≥40 ng/mL are associated with a significant reduction in the risk of many invasive cancers [105]. Similarly, another prospective cohort study stated that vitamin D levels ≥30 ng/mL are associated with a lower risk of tobacco-related cancers in smokers [106]. In observational trials of breast cancer, serum 25(OH)D levels were positively associated with lower rates of incident breast cancer particularly among postmenopausal women. These levels were inversely correlated with an aggressive form of the disease in pre-menopausal and postmenopausal women [107,108]. Furthermore, meta-analyses of cohort studies had shown better survival of breast cancer with high serum 25(OH)D status [109,110]. In colorectal cancer, a 19-year prospective study reported an inverse correlation between dietary vitamin D and calcium intake and the risk of colorectal cancer [111].

Clinical trials investigating the effect of vitamin D supplementation on the cancer incidence yielded contrasting results. In a randomized, double-blind, placebo-controlled, 7-year Women health initiative (WHI) trial, vitamin D and calcium supplementation (1 g calcium + 400 IU vitamin D3 daily) showed no effect on overall incidence of invasive cancers in postmenopausal women [112,113]. However, in post-hoc subgroup analysis, the supplementation was associated with reduced risk of total, breast, and colorectal cancers (in patients not using calcium and vitamin D supplements at baseline) and with decreased risk of melanoma (in women with history of non-melanoma skin cancer) [114,115]. Similarly, in another four-year, double blind, placebo-controlled randomized clinical trial, calcium and vitamin D supplementation (1.5 g calcium + 2000 IU vitamin D3 daily) did not lower the risk of all types of cancers (excluding non-melanoma skin cancers) [116]. A recently published double-blind, placebo-controlled RCT in colorectal adenoma has shown that the beneficial effect of vitamin D3 supplementation varies with VDR genotypes. This includes with reduced risk of advanced adenoma in individuals with VDR rs7968585 AA genotype and with increased risk of advanced adenoma in individuals with VDR rs7968585 GG/GA genotypes [117]. Meta-analysis of RCTs on vitamin D supplementation observed very little influence of vitamin D supplementation (400–1100 IU/day for two to seven years) on cancer incidence, but stated its significant role in reducing the total cancer mortality rate [118].

### 4.2. Diabetes

#### 4.2.1. Type 1 Diabetes (T1D)

Experimental studies have indicated the beneficial role of vitamin D in Type 1 Diabetes. Derakhshanian et al., have shown that vitamin D significantly improved fasting glucose, insulin, and IGF-1 in the type 1 diabetes (T1D) rat model [119]. Similarly, vitamin D treatment was shown to enhance the insulin secretion and to suppress the apoptosis of pancreatic β-cells in the T1D mice model [120]. Ysmail-Dahlouk et al., suggested that the beneficial effect of vitamin D on T1D could be mediated by its anti-inflammatory actions like up-regulation of IL-4, IL-10, arginase activity, and p-STAT6 and down-regulation of IFN-γ, IL-17, NO, and p-STAT4 levels [121]. In accordance with the previous study, Mao et al., have shown that calcitriol supplementation downregulated serum and urinary inflammation markers like TNF-α, IL-6, and ICAM-1 in T1D patients [122].

The observational studies investigating the relation between vitamin D and T1D are inconsistent. However, a meta-analysis of these studies suggests that vitamin D intake during early life is associated with lower risk of T1D [123,124,125]. The effect of vitamin D supplementation on pancreatic β-cell functions also varies across different studies and the literature about the role of vitamin D repletion on the glycemic control in T1D patients is scarce and shows contradictory findings [126,127]. Few clinical trials have observed that vitamin D and its analogs either preserved the β-cell function or led to a slow decline of residual β-cell function in children with T1D and adults with latent autoimmune diabetes [128,129,130,131]. In contrast, other studies did not find any role of vitamin D supplementation on β-cell functions in T1D patients [132,133]. An RCT studied the effect of vitamin D3 supplementation on Treg cells in T1D patients and reported an improved suppressor capacity of Treg cells in the treatment group (70 IU/Kg bodyweight of cholecalciferol daily) when compared to the placebo group [134].

#### 4.2.2. Type 2 Diabetes (T2D)

Similar to T1D animal studies, paricalcitol (a vitamin D analog) treatment in type 2 diabetes (T2D) rats significantly decreased the plasma glucose and insulin resistance by modulating the pancreatic oxidative stress and the inflammatory markers (like C-peptide, adiponectin, pancreatic IL-2, catalase, superoxide dismutase, glutathione peroxidase, and TNF-α) [135]. Elseweidy et al., showed that the administration of vitamin D in diabetic rats reduced the insulin-degrading enzyme and activated the insulin receptor phosphorylation, which improved the glycemic index and insulin resistance [136].

The results from observational studies suggest that a low vitamin D level increases the risk of hyperglycemia in diabetic and non-diabetic subjects [137]. Additionally, some clinical trials including meta-analysis showed that vitamin D supplementation significantly improves the glycemic control and the metabolic parameters in pre-diabetic and diabetic patients [138,139,140,141]. A similar positive effect of vitamin D supplementation was observed in gestational diabetic patients [142]. It was also shown that T2D subjects with specific genotypes (VDR-Cdx-2AA) respond better to vitamin D supplementation [143]. A recent meta-analysis of RCTs suggests that vitamin D supplementation improves the chronic low-grade inflammation in T2D patients by reducing the C reactive protein level, TNF-α level, and the erythrocyte sedimentation rate by enhancing the leptin level [144]. However, in several other studies, no effect of vitamin D supplementation on β-cell function, insulin sensitivity, or glycemic outcomes in pre-diabetic or diet-treated diabetic patients was observed [126,145,146,147,148]

### 4.3. Hypertension and Cardiovascular Diseases

#### 4.3.1. Hypertension

Renin-angiotension system (RAS) is a key regulator of blood pressure, electrolytes, and volume homeostasis. Its abnormal activation is a major risk factor for hypertension and cardiac disease. It has been demonstrated that 1,25(OH)_2_D3 regulates RAS by reducing renin expression in a VDR-mediated mechanism [149]. Similarly, Carrara et al., have shown that the restoration of a normal vitamin D level resulted in the inhibition of peripheral RAS system and improvement of endothelial function in hypertensive patients with low vitamin D [150]. Studies using renal arteries from hypertensive patients reported that calcitriol reduced the expression of the angiotensin-1 receptor in endothelial cells, which improved endothelial function and prevented reactive oxygen species (ROS) overproduction [151].

Many epidemiological studies have also shown an inverse association between serum vitamin D levels and the risk of hypertension [152,153,154]. However, most of the RCTs did not reciprocate similar findings and suggested no significant effect of vitamin D supplementation on blood pressure in hypertensive subjects [155,156,157,158]. However, the role of vitamin D supplementation in hypertension based on RCT studies cannot be completely negated as certain studies showed its positive effect in reducing blood pressure either in a particular-group of patients like type 2 diabetic subjects and dark-skinned hypertensive patients and as an adjuvant therapy in grade I-II hypertensive subjects [159,160,161].

#### 4.3.2. Cardiovascular Diseases

Molecular studies have indicated a beneficial role of vitamin D in cardiovascular diseases by regulating thrombosis, atherosclerosis, endothelial function, RAS, vascular calcification, and cardiac hypertrophy. It has been shown that vitamin D and its analogs prevent the thrombosis by down regulating pro-thrombotic factors like vascular smooth muscle cells derived tissue factor, PAR-2, plasminogen activator inhibitor-1, and thrombospondin and by up-regulating the anti-thrombotic factor thrombomodulin [162,163]. The anti-inflammatory action of vitamin D in endothelial cells was shown to prevent the initiation or the progression of atherosclerosis [164,165]. Furthermore, it was also suggested that vitamin D could prevent atherosclerosis by inhibiting the transformation of macrophages to foam cells and by inducing vessel relaxation [166,167,168]). The role of vitamin D in cardiac hypertrophy was shown in studies on VDR knockout mice and 25(OH)D-1-α-hydroxylase knockout mice, which displayed myocardial hypertrophy [169,170]. Vitamin D exerts this anti-hypertrophic effect in many ways such as by suppressing pro-hypertrophic calcineurin/ nuclear factor of activated T cells (NFAT)/ myocyte-enriched calcineurin-interacting protein 1 (MCIP1) pathway [171], by inhibiting cardiac RAS system [172], and by inhibiting cardiomyocytes proliferation [173]. The effect of vitamin D on vascular calcification is complex. It may increase the vascular calcification through its action on mineral metabolism and promotion of osteoblastic gene expression while it may reduce the vascular calcification through its interaction with fibroblast growth factor 23 (FGF-23) and klotho [168].

In observational studies, low vitamin D status was found to be associated with increased rates of cardiovascular risk factors, with more severe coronary artery disease, and a poor prognosis in patients with heart failure [174,175,176,177]. Additionally, some RCTs have indicated a beneficial effect from vitamin D supplementation as an adjuvant therapy on these latter conditions [178,179,180]. Recently, a meta-analysis showed an inverse relationship between serum 25(OH)D levels and events/mortality of cardiovascular diseases [181]. However, many RCTs did not find any obvious effect of vitamin D supplementation on cardiovascular risk factors in hypertensive patients, premenopausal women, or coronary artery disease subjects and did not show any effect of vitamin D supplementation on the markers of vascular function in myocardial infarction patients [155,182,183,184]. Similarly, treatment of postmenopausal women with calcium (1000 mg of elemental calcium daily) and vitamin D3 (400 IU daily) did not modify their coronary artery calcified plaque burden [185].

### 4.4. Auto-Immune Diseases

The immunomodulatory actions of vitamin D indicate its important role as a therapeutic agent in autoimmune diseases. It is suggested that vitamin D deficiency may promote autoimmunity by favoring the disproportionate production of Th17 and Th9 cells [186].

#### 4.4.1. Multiple Sclerosis (MS)

Vitamin D treatment in multiple sclerosis (MS) patients reduced the proliferation of CD4^+^ T cells and myelin basic protein specific T cells, decreased the frequency of IL-6 and IL-17 secreting cells, and promoted the development of IL-10 secreting cells and CD4^+^CD25^+^ T regulatory cells [60,187]. Furthermore, vitamin D is reported to specifically interact with HLA-DRB1*1501 haplotype (the strongest genetic risk factor of MS) and to influence its expression through VDRE. The latter is consistently expressed in homozygous carriers of HLA-DRB1*1501 [188].

Similarly, several epidemiological studies have shown the association of low serum vitamin D level and low intake of vitamin D with a higher risk of MS [189,190]. Many studies suggested that vitamin D supplementation, as an addition for therapy to IFN-β, shows a beneficial effect on MS and Relapsing-Remitting MS (RRMS) patients. Vitamin D treatment (including its analog, alfacalcidiol) in MS/RRMS patients resulted in a significant improvement in mental quality of life, reduction in MRI disease activity, and low frequency of relapses [191,192,193]. Additionally, the vitamin D-treated group had higher proportion of relapse-free patients compared to the placebo group [192]. It was also observed that vitamin D supplementation limits the titers of antibodies against the Epstein-Barr virus with the latter increased after the onset of the disease in MS patients [194]. In contrast to these positive findings, most reports suggest no effect of vitamin D on the clinical outcomes of the RRMS or MS patients [195,196].

#### 4.4.2. Systemic Lupus Erythematosus (SLE)

Hypovitaminosis D is common in systemic lupus erythematosus (SLE)patients and is usually associated with higher disease activity [197,198] pointing to a potential beneficial role of vitamin D supplementation on SLE patients. Furthermore, in juvenile SLE, cholecalciferol supplementation (50,000 IU/week) for 24 weeks significantly decreased the disease activity and also improved fatigue scores [199]. Similarly, in another study, vitamin D intake (2000 IU/day for 12 months) in SLE patients improved the inflammatory and hemostatic marker levels and reduced disease activity [200]. The beneficial effect of vitamin D on SLE may be due to its ability to expand Treg cells and to its capacity to reduce the frequencies of Th1 cells, Th17 cells, memory B cells, and auto-antibodies [201].

#### 4.4.3. Rheumatoid Arthritis (RA)

Molecular studies have suggested that vitamin D deficiency in rheumatoid arthritis (RA) stimulates the inflammatory response and promotes the osteoclast-mediated bone resorption [202]. Additionally, vitamin D treatment exhibited an anti-inflammatory response in RA by downregulating IL-6-and TNF-β and through enhanced differentiation of Treg and Breg cells. Furthermore, it has been demonstrated that vitamin D inhibited the osteoclastogenesis and bone resorption in RA by downregulating RANKL and upregulating its decoy receptor osteoprotegerin [202,203]. Epidemiological studies suggest that vitamin D deficiency is common in RA patients. However, its relationship with the disease activity is not clear [204,205]. Reports on the influence of vitamin D supplementation on RA are not consistent. While two open blind studies observed a significant effect of vitamin D repletion on pain relief and disease activity [206,207], others did not demonstrate any role of vitamin D supplementation on the clinical parameters of RA patients [208].

#### 4.4.4. Auto-Immune Thyroid Diseases (AITD)

Animal studies have shown that the vitamin D treatment either prevents the development of experimental autoimmune thyroiditis or improves the inflammatory status of the gland by ameliorating the structural changes and normalizing the auto antibody production and the cytokines levels in experimental autoimmune thyroiditis animal models. Vitamin D treatment lowered IFN-γ and IL-12 and increased IL-4 and IL-10 levels [209,210]. In addition, many observational studies have shown a significant association of vitamin D deficiency with the risk of auto-immune thyroid diseases (AITD) [211,212,213] and with anti-thyroid antibodies and abnormal thyroid function [214,215]. However, some reports did not find any link between vitamin D deficiency and the risk of AITD including Hashimoto thyroiditis [216,217,218]. Clinical trials investigating the effect of vitamin D supplementation in AITD are limited and inconclusive. While some of them suggested the significant reduction in thyroid auto-antibodies levels (thyroperoxidase autoantibody (TPO-Ab) and thyroglobulin autoantibody (TgAb)) and the improvement in thyroid function tests (thyroid-stimulating hormone (TSH) and Free Thyroxine (FT4) levels) with vitamin D supplementation in AITD patients [219,220], others showed no effect of vitamin D treatment on thyroid function and autoimmunity in AITD patients [221].

#### 4.4.5. Systemic Sclerosis (SSc)

Vitamin D is supposed to mediate a beneficial effect in SSc by decreasing the fibrosis in different tissues. It was observed that VDR expression is decreased in lesional skin, in fibroblasts of systemic sclerosis (SSc) patients, and in fibroblasts of animal models of SSc [222]. It was also demonstrated that the downregulation of VDR in SSc patients’ fibroblasts and in SSc animal models’ fibroblasts occurred in a TGF-β-dependent manner. Experts suggested that impaired VDR signaling either due to low VDR expression or low vitamin D level might lead to hyperactive TGF-β signaling and aberrant fibroblast activation in SSc [222]. Additionally, paracalcitol (an analog of vitamin D) treatment in animal model of SSc was found to restore VDR signaling and to decrease the pro-fibrotic effects of TGF-β on fibroblasts.

Hypovitaminosis D seems to be common in SSc patients, which was reported in many observational studies [223,224,225,226]. In addition, several case control studies including a meta-analysis study have shown significantly lower vitamin D level in SSc patients compared to controls [227,228,229]. Some studies have reported a correlation between vitamin D deficiency and clinical features of SSc patients like skin, cardiac, and lung involvement as well as peripheral vascular, kidney, and gastrointestinal damage [227,230,231,232]. The association of vertebral fracture and osteoporosis with lower levels of vitamin D was also reported in patients with SSc [232]. There are few contradictory studies, which either found no difference in vitamin D levels of SSc patients and controls or showed no association of vitamin D with clinical parameters of SSc [229,233]. Clinical trials on supplementation of vitamin D in SSc patients are very scarce. While one study showed a beneficial effect of 1,25(OH)_2_D3 supplementation in SSc patients [234], another study was inconclusive due to a very small sample size [235].

### 4.5. Dermatological Diseases

Vitamin D, which can be synthesized in the skin, regulates several physiological processes in the skin-like proliferation, differentiation, and apoptosis of keratinocytes and maintenance of normal skin barriers and immune system. Vitamin D deficiency has been linked to many skin pathologies. Some of these pathologies are described below.

#### 4.5.1. Atopic Dermatitis (AD)

Atopic dermatitis is characterized by a skin barrier dysfunction and immune dysregulation. Studies have suggested that vitamin D may exert a beneficial effect in atopic dermatitis (AD) by normalizing the altered Th1 and Th2 cytokines (like IL-2, IL-4, IL-6, and IFN-γ), upregulating AMP in the skin, and improving the skin barrier function [236,237]. Furthermore, vitamin D treatment can dampen the enhanced IgE response in AD by lowering the IgE production by human B cells and suppressing IgE-mediated mast cell activation [238]. Many case-control studies have shown a lower level of vitamin D in AD subjects compared to controls and the association of vitamin D deficiency to a more severe course of the disease [239,240,241]. In a prospective cohort study, it was observed that vitamin D-deficient individuals have an increased likelihood of developing AD [242]. Additionally, many RCT have observed a significant improvement of disease severity with vitamin D supplementation [243,244,245].

#### 4.5.2. Psoriasis

Vitamin D, through its anti-inflammatory action, has a beneficial effect in Psoriasis. Molecular studies have shown that vitamin D treatment in psoriatic lesions inhibits pDC capacity to induce T-cell proliferation and IFN-γ secretion, suppresses the enhanced Th17 pathways, and upregulates the expression of late cornified envelop genes (*LCE3B* and *LCE3C*) [246,247,248]. Vitamin D deficiency seems to be common in psoriatic patients, which was reported in many studies [249,250]. Vitamin D derivatives, used topically, are a mainstay in the treatment of psoriasis. It has been shown that the topical use of vitamin D (calcitriol twice daily for eight weeks) and its analogs (paricalcitol once daily for 12 weeks, maxacalcitol once daily for eight weeks, and becocalcidiol twice daily for eight weeks) is an effective and safe therapy for mild to moderate plaque psoriasis [251,252,253,254]. Similarly, maxacalcitol topical ointment twice daily for eight weeks was found to be effective in the management of palmoplantar pustulosis [255]. Many open-labelled clinical studies have also shown the efficacy and safety of vitamin D analogs in the treatment of facial, nail, and scalp psoriasis [256,257,258].

#### 4.5.3. Vitiligo

The protective effect of vitamin D in vitiligo is thought to be mediated by its anti-oxidative and anti-apoptotic effect on melanocytes. Vitamin D treatment is reported to prevent apoptosis of melanocytes, to enhance the melanogenesis and the tyrosinase content of melanocytes, and to reduce the oxidative stress by decreasing intracellular ROS overproduction, enhancing superoxide dismutase activity, and decreasing the level of malondialdehyde [259,260]. Many studies have shown that vitiligo subjects are either deficient or insufficient in vitamin D, but when compared to controls, the difference is not always statistically significant [261,262,263]. Clinical studies investigating the effect of topical application of vitamin D and its analogs are contradictory. Some studies have shown the efficacy and safety of calcipotriol or tacalcitol for the treatment of vitiligo alone or as combination therapy [264,265,266] while others did not find any significant change in re-pigmentation or lesion size in vitiligo patients [267,268].

Recently, a meta-analysis of the studies on the off-label use of topical vitamin D in the treatment of dermatological diseases has recommended the usage of vitamin D for treating various ichthyoses, morphea, pityriasis alba, prurigo nodularis, and polymorphous light eruption and in vitiligo as combination therapy alongside corticosteroids and phototherapy [269].

### 4.6. Polycystic Ovarian Syndrome (PCOS)

The vitamin D deficiency is common in polycystic ovarian syndrome (PCOS) [270,271,272] and the two recent meta-analysis of clinical trials point to the beneficial role of vitamin D in PCOS patients. These meta-analyses observed an improvement in follicular development, menstrual cycle regulation, serum PTH, and triglycerides levels [273,274]. Similarly, vitamin D and calcium co-supplementation for eight weeks was found to positively impact the biomarkers of inflammation and oxidative stress in PCOS [275].

Vitamin D repletion in PCOS women was shown to significantly lower the serum VEGF levels or reduce the TGF-β bio-availability. Both of them were separately linked to improvements associated with clinical parameters of PCOS [276,277]. However, there are some clinical studies, which showed no significant effect of vitamin D supplementation on anthropometric or metabolic parameters in PCOS women [278,279].

## 5. Conclusions

Apart from its effect on the bone mineral metabolism, vitamin D exhibits an extensive effect on multiple biological processes in humans like cellular proliferation, differentiation, apoptosis, and immune regulations. Epidemiological and molecular studies have indicated the role of vitamin D in various health disorders ranging from skin diseases, cardiovascular disorders, cancers, the polycystic ovarian syndrome, autoimmune disorders, and many more. However, the results of randomized clinical trials do not echo the positive impact of vitamin D in many human diseases with similar intensity. This could be due to limited sample sizes, differences in ethnicity, variations in vitamin D dosage, and discrepancies in unmeasured confounding principles across different clinical trials. However, inconsistencies between the findings of molecular studies, observational studies, and randomized clinical trials in many human disorders also raise questions about the role of vitamin D whether it lies in causal pathways of human disorders or whether low vitamin D levels are only a phenomena in human diseases. Given the widespread effect of vitamin D on key biological processes, it seems unfair to consider vitamin D as only a marker of ill health. However, strong evidence supporting the causal relationship between vitamin D and non-skeletal human disorders is lacking. In the future, well-designed multi-centric clinical trials in large cohorts may provide definitive answers about the causal effect of vitamin D in human diseases and about the effective and safe usage of vitamin D/its analogs as therapeutics in complex disorders.

## Abbreviations

AMP	Anti-microbial peptide
DC	Dendritic cell
ng	Nanogram
nmole	Nanomole
RCT	Randomized clinical trial
Th cells	T helper cells
UVB	Ultraviolet B
VDR	Vitamin D receptor
